# A Non-Invasive Phase Sensor for Permittivity and Moisture Estimation Based on Anomalous Dispersion

**DOI:** 10.1038/srep28626

**Published:** 2016-06-27

**Authors:** Omar Siddiqui, Rashad Ramzan, Muhammad Amin, Omar M. Ramahi

**Affiliations:** 1 College of Engineering, Taibah University, Madinah, Saudi Arabia; 2 Department of Electrical Engineering, UAE University, Al-Ain, United Arab Emirates; 3 Department of Electrical and Computer Engineering, University of Waterloo, Waterloo,ON, Canada

## Abstract

The traditional microwave resonance sensors are based on the measurement of the frequency shift and bandwidth of a resonator’s *amplitude* spectrum. Here we propose a novel sensing scheme in which the material properties are estimated by determining the changes in the *phase* spectrum of an anomalous-phase resonator. In the proposed phase sensing, we exploit the unique double phase reversal which takes place on the edges of the anomalous dispersion region as a signature to detect the resonance. We show that with the phase sensing, a significant reduction in detection errors compared to the traditional sensing can be obtained because of the noise immunity offered by the phase detection and also due to the strong dispersive phase response that reduces the sensor’s dependence on the external environment. We also show that the bandwidth determination procedure of the resonance which is needed to characterize the sample losses is significantly simplified. The concept of phase sensing is shown by devising an experimental microstrip open stub resonator whose frequency response lies in the anomalous dispersion region. The dielectric characteristics of the samples placed on the stub are extracted from the resonant frequency and the slope of the phase response. We also demonstrate that the changes in moisture levels can also be detected by utilizing the phase sensing method.

The microwave sensors can be grouped into two broad categories i.e., the resonant and non-resonant methods^1^. Resonance based microwave sensing methods have been traditionally applied in situations where dielectric properties are required in a narrow range of frequencies^2^,^3^,^4^,^5^,^6^,^7^. For example, in the dielectric resonator methods, the sample is fabricated in specific shapes and are placed between metallic plates^2^. The relative permittivity is calculated from the spectral shifts in the resonant modes and the loss tangent is determined from the quality factor (Q) of the resonance. While in the cavity perturbation resonance methods, a dielectric is introduced in an electromagnetic resonator such as a rectangular waveguide^3^,^4^. The dielectric properties are then estimated from the properties of the perturbed resonant fields. The resonator methods have also been employed in other sensing schemes such as glucose^8^ and moisture level detection^9^. Although the resonant methods are more accurate and computationally less-exhaustive, they are inherently narrow band and hence a separate setup for each discrete frequency is required. On the other hand, the non-resonant measurement techniques are employed where a generalized view of material properties is required over a broad range of frequencies^10^,^11^,^12^. Hence they require accurate calibration of the measurement setup over a wide spectrum, and involve considerably more computational resources with mathematical rigour. In pursuit of achieving higher sensitivities and compact designs, metamaterial-inspired detection techniques have been introduced to the field of dielectric sensing^13^,^14^,^15^. In an energy-tunneling sensor, enhanced sensitivities were obtained by allowing the electromagnetic field to concentrate in narrow waveguide channels^13^,^14^. The metamaterial-based RF tag sensors that employ phase modulation coding schemes to identify different parameters have been found particularly useful since the sensed energy is independent to the measured quantity^15^.

To date, all of the reported microwave material characterization resonance-based methods utilize the amplitude spectrum of either the transmission or reflection coefficient for the electromagnetic parameter extraction (see^1^,^16^ and the references therein). The obvious reason is the fact that the amplitude response of a resonator contains a distinctive feature i.e. the resonance dip (or rise) from which the spectral information and thus the material properties can be extracted. A distinctive resonance feature, however, is absent from a usually encountered phase spectrum. An exception to the normally phase characteristics is the spectrum of the anomalous dispersion region which is characterized by a double reversal of the phase-slope in the vicinity of the resonance^17^,^18^,^19^,^20^. Therefore, the dielectric characterization based on the anomalous dispersive resonance would be less ambiguous as it would consist of two distinctive signatures i.e., an amplitude dip accompanied by a phase-slope reversal. In a recent research work^21^,^22^, we have exploited the anomalous dispersive characteristics of a microstrip circuit in an inverse complex permittivity determination problem. The complex permittivity inversion from the anomalous phase was possible because of the highly dispersive nature of the transmission response of the microstrip structure. The Kramers-Kronig relations were thus applied on the phase spectrum to determine the dielectric characteristics of the microstrip substrate.

In this paper, we exploit the anaomolous dispersion phenomenton for non-invasive and non-destructive *sensing* by introducing the dielectric sample in an anomalously dispersive microstrip cavity and then determining the spectral location and slope of the shifted phase spectrum. The relative permittivity (*ε*_*s*_) and the loss tangent (*tanδ*) are then extracted from the resonance frequency and phase-slope by comparing them with pre-calculated calibration curves obtained from full-wave numerical simulations. With proper calibration, the proposed method can be readily extended to other types of sensors such as as humidity and glucose sensors. In the next section, we first present the details of the phase sensing microstrip structure followed by a comparison between the amplitude and phase sensing, and finally experimental results of dielectric and moisture sensing.

## Results

### The Microstrip Resonant Structure

**Figure 1:**
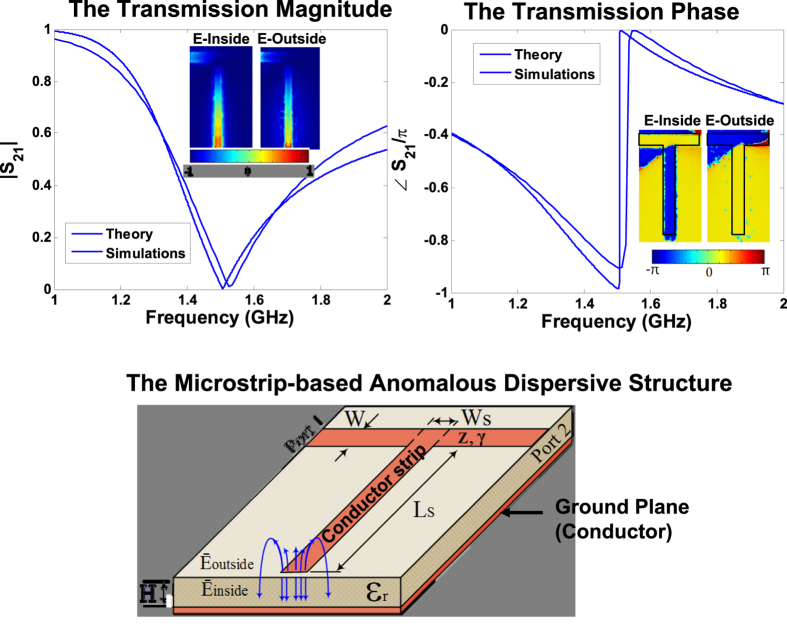
A Microstrip-based Anomalously Dispersive Cavity and associated Magnitude and Phase transmission responses. The theoretical responses are calculated by applying Eq. 1 and the simulated responses were found by using COMSOL full-wave simulator. The small difference between the theoretical and simulated resonant frequencies is due to the approximations in Eq. 1. The distributions of the resonant electric field’s magnitude and phase are shown in the insets of the transmission coefficients’ plots.

A representative microwave anomalously dispersive structure is depicted in Fig. 1, which consists of a through microstrip line of length *d* loaded by an open circuit stub of admittance *Y*_*s*_. The figure also shows the plots of the full-wave simulations of the transmission response performed in the electromagnetic simulator COMSOL along with the analytical model given by the following mathematical expression:


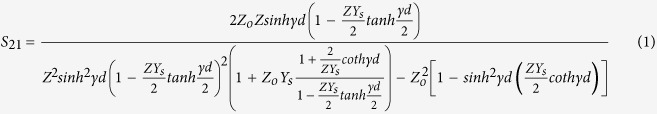


where *γ* and *Z* are the complex propagation constant and the characteristic impedance of the microstrip line and *Z*_*o*_ is the port impedance. The anomalous dispersion region is identified by the increased transmission losses and the reversal of the phase slope. The inherent resonance frequency (1.53 GHz in this example) depends on the open stub length *L*_*s*_ and the effective permittivity of the microstrip line *ε*_*e*_ and is approximately given by:


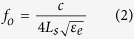


The strongly resonant magnitude and phase spectra are connected through the well-known Kramers-Kronig relations or in case of electrical circuits with the subsequent extension to electronic circuits by Bode^23^,^24^. Hence the material constituting the microstrip’s substrate can be completely characterized by the phase measurement in the anomalous dispersion region^21^,^22^. In particular, the resonant frequency corresponds to the real part of the dielectric constant (*ε*_*r*_) and the slope of the phase is related to the loss tangent (tan*δ*) of the substrate material.

### A Comparative study of Amplitude and Phase Sensing

**Figure 2:**
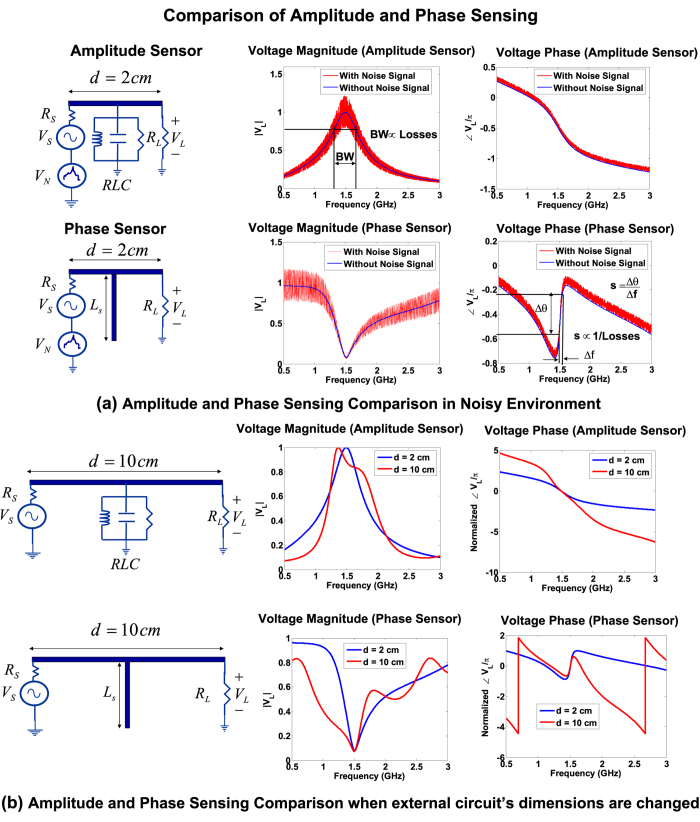
The amplitude sensor is adversely affecred by additive noise and changes in external sensor circuitry. The phase sensor is immune to these adverse effects. (**a**) Performance Comparison of the amplitude and phase sensors in a noisy environment. The bandwidth of the amplitude spectrum of the amplitude sensor is significantly affected when an additive noise signal is injected at the input. When the noise signal is injected in the phase sensor, the phase slope of the anomalous dispersion (which corresponds to the loss characterization) is minimally affected. (**b**) Effect of external circuit changes on the performance of the amplitude and phase sensors. The increase in the lossy feedline lengths changes the bandwidth of the amplitude response. The same effect does not produce any significant change in the phase slope of the anomalous dispersion region.

In this sub-section, we compare the performance of the *phase* sensor (presented in the previous sub-section) with a representative *amplitude* sensor which can be designed by loading a transmission line with a parallel RLC resonator. Figure 2 shows the two types of sensors along with their respective frequency responses, obtained by solving Eq. 1. The material characterization involves the determination of the resonant frequency and the bandwidth of the resonance. As shown in Fig. 2, the sample’s loss characterization for the amplitude sensor is done by measuring the bandwidth of the amplitude spectrum which requires a full scan of the resonance tip between the two half-power frequencies. On the other hand, the procedure of bandwidth determination is much simplified in the phase sensing where the losses are characterized by determining the slope of the phase spectrum which only requires a two-point measurement. Also note that the frequency response of the amplitude sensor contains only one resonance signature in the amplitude spectrum. However, the phase sensor contains a second resonance signature in its phase spectrum. Hence we anticipate a more accurate and computationally less exhaustive procedure of resonance and bandwidth characterization using the proposed sensing method.

Next, we inject an additional white noise electrical signal *V*_*N*_ to the input of the sensors, so that the total input voltage is given by:


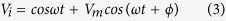


where the amplitude *V*_*m*_ and the phase *ϕ* are uniformly distributed between 0 and 200 *mV* and between 0 and 180° respectively. As shown in 2a, the bandwidth of the amplitude sensor response is considerably affected by the noise. On the other hand, on account of the strong dispersive nature of the anomalous dispersive phase and the inherent noise fighting capability of the phase detection, the slope of the anomalous dispersive phase is noticeably not affected by the white noise. Therefore, we anticipate our proposed sensor to detect material properties with much more accuracy compared to the traditional approaches in noisy environments such as furnaces, automobile engines and in space missions.

To observe the effect of external circuitry on the sensor performance, we increased the lengths of the transmission lines that feed the sensors by five times. Because of the presence of propagation losses in the transmisison lines, given by *e*^−*αd*^ (*α* = 0.1 *Np*/*m*), a visible increase of bandwidth is observed in the amplitude response detected by the amplitude sensor. Interestingly, the slope of the phase spectrum in the anomalous dispersion region only changes from 1.5° to 1.3° per MHz. This minimal slope change is the result of the highly dispersive nature of the microstrip structure and leads to a much smaller error in the loss tangent characterization of the sample under test. Hence, we also expect the proposed phase to demonstrate a much superior performance than its traditional amplitude-based counterparts in environmental changes that affect the external sensor circuitry.

### The Sensing Methodology and the Calibration curves

The non-invasive sensing principle can be explained by considering Fig. 1 and noting that the microstrip transmission line do not support a pure transverse electromagnetic mode (TEM) since the dielectric (*ε*_*r*_) does not fill the region above the strip. Therefore, in addition to the dominant electric fields that exist between the ground and the strip, a fraction of the field directed perpendicular to the strip also fills the region above the strip in air. Therefore, effectively, the resonant cavity is composed of the substrate and the space above it that contains the electric field fringes^25^. As depicted in the Fig. 1, these outside-the-substrate resonant electric fields are considerably enhanced on the edge of the open-stub during the anomalous dispersive resonance and hence could be exploited in material detection and sensing. However, away from the open-stub edge, the electric field inside the substrate are larger in magnitude. This behavior is consistent with the propagation mechanics of the dominant quasi-TEM. The resonance phase plots depict a 180° shift between the inside and outside the substrate fields since both originate from the common conducting strip and flow in the opposite directions. If a dielectric sample is placed on the open stub, it will perturb the air fields thereby increasing the effective dielectric constant experienced by the open-stub, resulting in the redshift of the resonance. The resonance shift is also accompanied by a relative change in the anomalous phase slope which corresponds to the losses in the sample. A numerical calibration procedure can be implemented to estimate the dielectric constants and the loss tangents taking into account the relative shift of the two resonance parameters from their inherent values (given by Equations 1 and 2).

**Figure 3:**
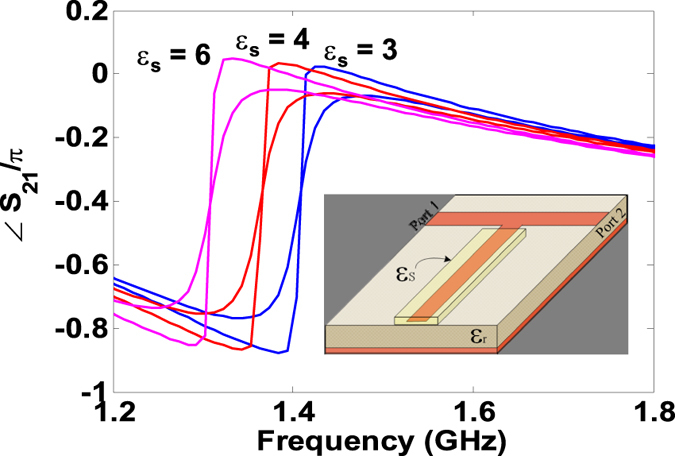
Phase simulations show the frequency shift and phase-slope changes in the anomalous dispersion region with the change in the dielectric parameters of the sample placed on top of the open-stub. The larger phase slope indicates a loss tangent of 0.001 and the smaller phase slope corresponds to a loss tangent of 0.1.

**Figure 4:**
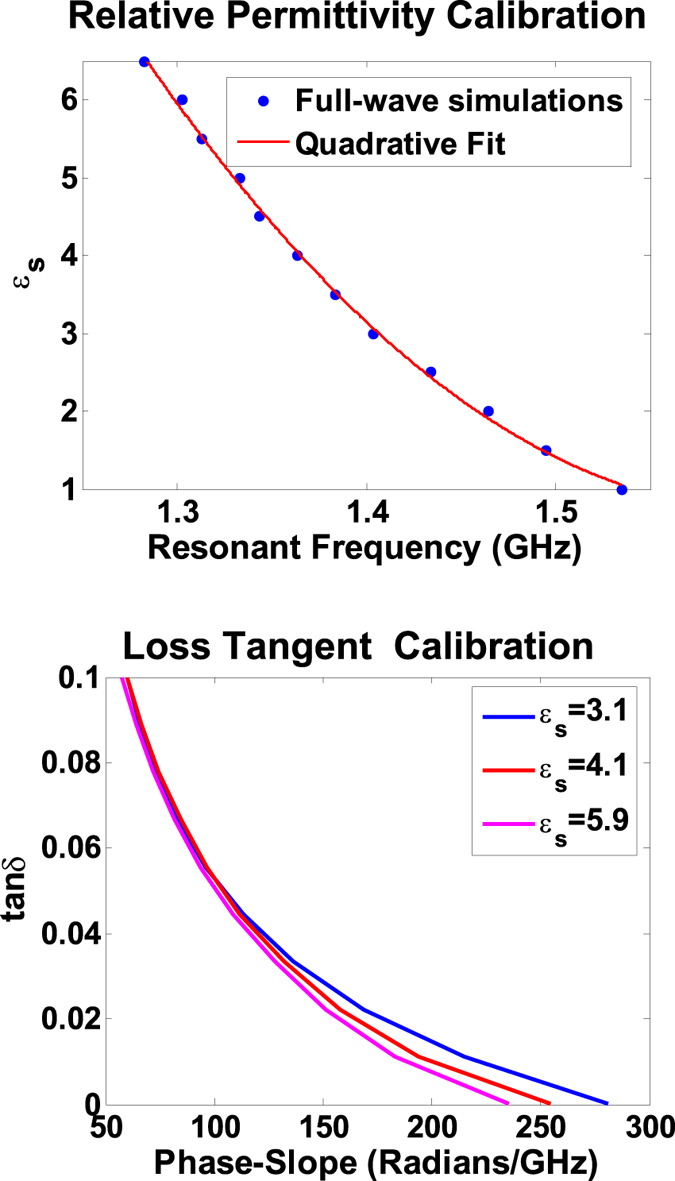
The calibration curves generated by full wave simulator COMSOL to calculate the complex dielectric constant of a sample. The top curve demonstrates the relation between the resonant frequency and the relative permittivity of the sample. The bottom curve shows the effect of change in sample’s loss tangent on the slope of the anomalous phase.

Consider the numerical simulation of the sensing procedure in which the dielectric samples of varying complex dielectric constant are placed on the open-circuit microstip stub. For example, as shown in Fig. 3, the placement of a sample with *ε*_*s*_ = 3.1, results in the resonance redshift from 1.53 GHz to 1.4 GHz. More interestingly, an increase in sample’s loss-tangent renders a decrease in the slope of the anomalous dispersion region which corresponds to the increase in the resonant bandwidth. Hence the bandwidth characterization which is needed to estimate the the loss-tangent (or the Q-factor) only require a two point phase measurement. The effect of resonance shift and slope change (observed in Fig. 3) can be summarized in the two calibration curves generated by COMSOL and are depicted in Fig. 4. A dielectric material sample can thus be completely characterized by measuring the resonance frequency shift and the slope of the anomalous dispersive region.

### Practical Determination of Complex Dielectric Constants

**Figure 5:**
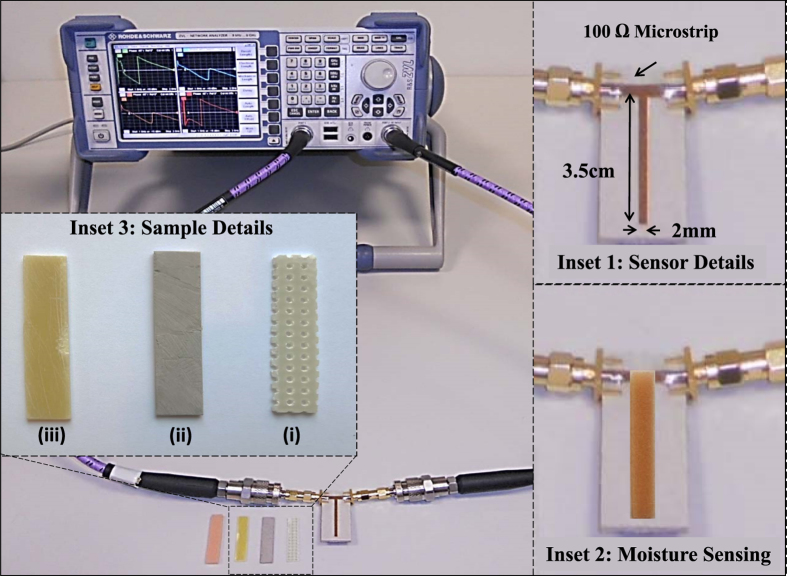
The experimental set up of the sensing technique based on anomalous dispersion. The inset 1 shows the geometrical details of the sensor and moistened sponge placed on the open-stub. The inset 2 shows the sensor with the moistened sponge. The inset 3 shows different samples (i) Air-depleted FR2 (ii) FR4 (iii) Rogers 6006.

**Figure 6:**
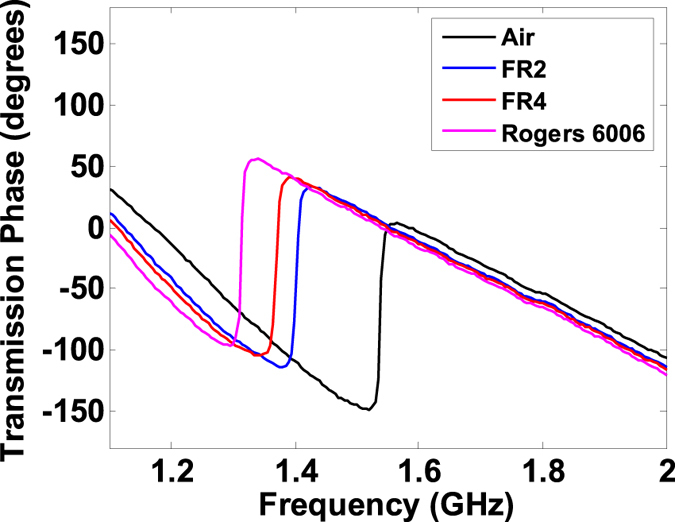
Transmission Phase spectra when samples are placed on the sensor.

The experimental set up of the proposed sensing technique is depicted in Fig. 5. Three dielectric materials i.e. the perforated- FR2 (used in pre-formed circuit boards), FR4, and Rogers 6006 were cut into rectangular 3.7 mm × 1 cm samples, as depicted in the inset of Fig. 5. The nominal values of dielectric constants (*ε*_*s*_) are given by 3.5, 4.5, and 6.2 and the *tanδ* are given by 0.01, 0.02, and 0.002 in the order in which the samples are mentioned above. The samples were sequentially placed on the open stub of the microstrip structure and transmission phase characteristics were measured. The phase spectra of the three samples are provided in Fig. 6. The relative permittivities and the loss tangents are extracted from the calibration curves given in Fig. 4 and are summarized in Table 1. Note that the extracted values of *ε*_*s*_ are within 10% of the actual values. As shown in the table, both the relative permittivity and loss tangents are under-predicted mainly due to the presence of air gaps between the strip and the sample (a well-known phenomenon in open ended probes^16^). To improve the numerical model and hence to obtain realistic extracted values, a small air gap was added between the sample’s edges and the circuit board while keeping the conductive strip intact with the sample. It was found after the calibration procedure using the COMSOL simulator that a 16 *μ*m gap is required for reasonably accurate permittivity predictions. As shown in the last column of the Table 1, both the extracted parameters are now within 5% of the nominal values. With better numerical models that include the other fabrication details such as connectors and other fabrication imperfections more accurate predictions are possible.

### Humidity and Moisture Sensing

**Figure 7:**
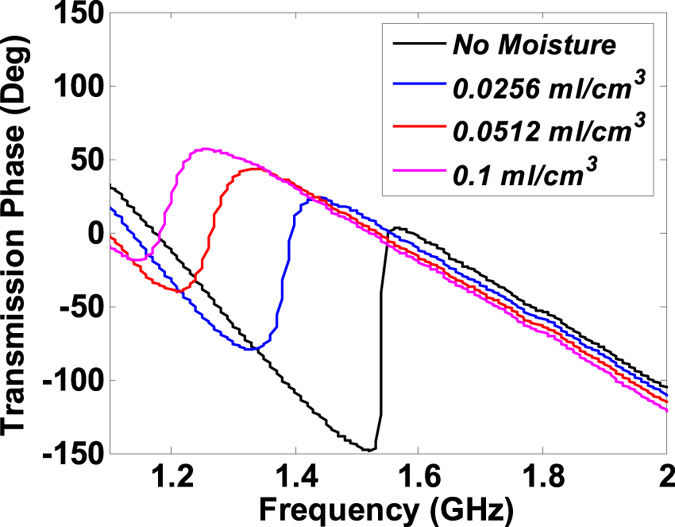
Phase spectra show resonance shift and slope changes when moisture is added to the sponge placed over the sensor.

**Figure 8:**
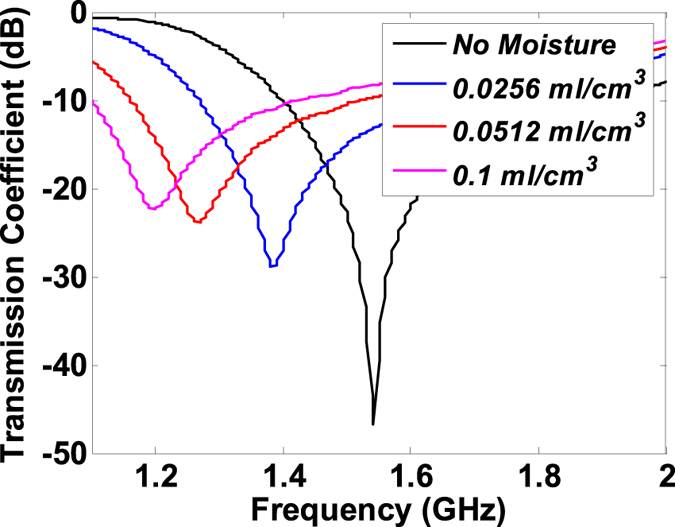
Amplitude spectra show decrease in transmission and the resonance shift when moisture is added to the sponge. Note that the phase spectrum is enough to characterize the moisture. Amplitude spectrum is given here to emphasize the relevance of the Kramers-Kronig relations.

The proposed anomalous dispersive sensor can be used to detect changes in environmental moisture or moisture pockets present in materials such as wood. To illustrate the moisture detection, a rectangular spongy material was placed on the anomalous dispersion sensor (Fig. 5) and water content was added to it by means of a dropper. Since water has a very large dielectric constant (approximately 80 at room temperatures), the addition of even a small amount of moisture content results in a considerable increase in the effective permittivity of the moistened sponge. For example, a large red-shift of 1.3 GHz is noted in Fig. 7 with a moisture content of 0.0256 *ml*/*cm*^3^. The increase moisture content also leads to more attenuation or an increase of the sponge’s loss tangent which translates to the decrease in the phase-slope. As more moisture is added to the sponge, the resonance frequency further shifts to the lower values accompanied by the lower slopes of the anomalous dispersive phase responses. The correspondence between the magnitude and phase response, as predicted by the Kramers-Kronig Theory, can be observed in the associated magnitude response 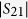 given in Fig. 8 where the amplitude dip shifts to lower frequencies and lower magnitudes with the increase in moisture.

## Discussion

We presented a non-invasive and non-destructive microwave resonance sensor based on the principle of phase sensing in the anomalous dispersion frequency region. We have shown the superior performance of the phase sensor over the traditionally implemented amplitude sensors through simulations and experiment. We have shown experimentally for the first time that by only measuring the *phase* response of the sensor, the complex permittivity or the moisture content of material samples can be estimated. We have demonstrated that the loss-tangent estimation only requires two phase measurements in the anomalous dispersion region. In the contemporary amplitude-based resonance sensing, on the other hand, the loss tangent estimation requires the bandwidth (or Q-factor) characterization which requires the measurement of the complete transmission dip over the resonance spectrum^5^. The dielectric constant of the sample has been shown to be proportional to the resonance shift of the sensor’s anomalous dispersion region while the loss-tangent (*tanδ*) behaves inversely with the phase-slope. The estimated values of the two quantities were obtained by comparing the experimental resonance frequency and phase-slopes with calibration curves numerically generated using the full-wave COMSOL simulator. Three commercially available dielectric samples (FR4, FR2, and Rogers 6006) were tested and the estimated dielectric constants were found to be within 5% of their known values. Note that similar to the other open-ended sensors, both the dielectric constants and loss tangents were under-predicted because of the presence of an air gap between the open-stub and the sample. Furthermore, we demonstrated experimentally that placement of a lightly moistened sponge (with moisture content 0.0256 *ml*/*cm*^3^) results in a 1.3 GHz resonance shift.

## Method

### Simulation Method

The Numerical simulations were conducted using the full-wave electromagnetic simulator COMSOL which is based on the Finite Element method. In the simulation setup, the microstrip structure of Fig. 1 is terminated in perfect electric boundaries towards the bottom side (ground plane) and by the radiation boundaries on the remaining sides. The calibration curves (Fig. 4a) are obtained by varying the real part of the sample’s dielectric constant (*ε*_*s*_) in the simulation setup and recording the resonant frequencies for each sample. Fig. 4b curve is obtained by varying the loss tangent of different samples (tan*δ*) and calculating the slopes of the anomalous phase. The complex dielectric constants of the dielectric materials given in Table 1 were obtained from the manufacturer’s data sheets.

### The Experiment

The prototype PCB, shown in Fig. 5, was designed using Agilent ADS based on the Rogers 6002 substrate which has a dielectric constant of 2.94 and a loss tangent of 0.0012. The designed PCB was manufactured using the MITS AUTOLAB milling machine which had a trace width accuracy of better than 100 *μ*m. To handle the soft Rogers 6002 material, the machine spindle speed and the bit diameter were carefully selected to avoid any material wrapping around the drill bit which could have affected the dimensional accuracy of the printed lines. The transmission characteristics were measured by the Rohde and Schwarz ZVL13 Vector Network Analyzer. The manual two port full calibration (through, open, short, and match) was done before the measurements. The three solid samples (FR2, FR4, and Rogers 6006) as depicted in Fig. 5 (inset 3) were sequentially placed on the microstrip PCB and the resonance shift and the change in anomalous phase was measured. The measured parameters were compared with the calibration curves (Fig. 4) to estimate the complex dielectric constants tabulated in Table 1. In the next round of the measurements, we placed the moistened sponge with moisture contents from 0.1 ml/cm^3^ to 0.025 ml/cm^3^ and the change in the both phase and magnitude of the transmission coefficients were measured.

## Additional Information

**How to cite this article**: Siddiqui, O. *et al*. A Non-Invasive Phase Sensor for Permittivity and Moisture Estimation Based on Anomalous Dispersion. *Sci. Rep.* **6**, 28626; doi: 10.1038/srep28626 (2016).

## Figures and Tables

**Table 1:** Estimation of the *ε*_*s*_ and *tanδ*.

Medium	Measured Parameters		Extracted Values (Fig. 3 Configuration)		Extracted Values (With Numerical Air Gap)	
	*f*_*r*_ (GHz)	Phase Slope (rad/GHz)	*ε*_*s*_	*tanδ*	*ε*_*s*_	*tanδ*
Air	1.53	288	1.0	0		
Perforated FR2	1.40	225	3.1	0.005	3.3	0.01
FR4	1.36	190	4.1	0.0125	4.3	0.02
Rogers 6006	1.3	240	5.9	0.0001	6	0.0019
